# Characteristics of anaphylaxis patients who visited emergency departments in Korea: Results from a national emergency department information system

**DOI:** 10.1371/journal.pone.0266712

**Published:** 2022-04-29

**Authors:** Mi-Hee Lee, Eui-Jeong Roh, Yu-Mi Jung, Youngmin Ahn, Eun Hee Chung

**Affiliations:** 1 Department of Pediatrics, Incheon Medical Center, Incheon, Republic of Korea; 2 Department of Pediatrics, Chungnam National University Hospital, Daejeon, Republic of Korea; 3 Medical Record Team, National Medical Center, Seoul, Republic of Korea; 4 Department of Pediatrics, Eulji General Hospital, Eulji University School of Medicine, Seoul, Republic of Korea; 5 Department of Pediatrics, Chungnam National University College of Medicine, Daejeon, Republic of Korea; Seoul National University College of Medicine, REPUBLIC OF KOREA

## Abstract

**Background:**

Anaphylaxis is an allergic disease with fatal respiratory or cardiovascular symptoms that require immediate emergency treatment. We aimed to understand the characteristics and frequency of emergency department (ED) visits of patients with anaphylaxis in Korea.

**Methods:**

Between 2007 and 2013, using data from 147 ED from the National Emergency Department Information System in Korea, we retrospectively evaluated patients with a primary diagnosis of anaphylaxis.

**Results:**

During the study, a total 23,313 patients visited the ED due to anaphylaxis. The number of patients with anaphylaxis who visited the ED increased from 3.0 per 100,000 population in 2007 to 11.6 per 100,000 population in 2013 (*P*<0.001). Overall, the frequency of anaphylaxis emergency department visits increased by 1.24 times each year (95% CI 1.23–1.25). The risk of visiting ED due to anaphylaxis by population-based age-specific group was highest in the 60–69 years old (OR 2.30, 95% CI 1.96–2.70). Deaths from anaphylaxis increased by 1.35 times per year (95% CI 1.13–1.62). The causes of anaphylaxis were unknown (80.8%; 95% CI 80.35–81.38), drugs (8.9%; 95% CI 8.47–9.24), food (4.1%; 95% CI 3.87–4.39), bees (3.2%; 95% CI 3.02–3.48) and arthropods (2.3%; 95% CI 2.11–2.48). In 2009, drugs were the most common cause of anaphylaxis in November (35.5%), followed by food in May (15.5%) (*P*<0.001). Between July and September, stings from insects were the most common causes (*P*<0.001). By age, food was the most common cause in children aged <6 years (7.6%, <12 months; 9.0%, 1–6 years) and drugs in those aged ≥7 years. The 7-year overall mortality rate was 0.104 case per 1,000,000 population; men accounted for 77.8% of the deaths. By region, the number of cases was the highest in metropolitan areas, Gyeonggi and Seoul; however, the number of anaphylaxis cases per 100,000 population was the highest in Jeju and Gangwon.

**Conclusion:**

Based on ICD-10 codes, the number of ED visits due to anaphylaxis is increasing in Korea, and the incidence of anaphylaxis varies by region, season, and age.

## Introduction

Anaphylaxis is a fatal respiratory or cardiovascular condition and is a medical emergency that requires immediate treatment. As the occurrence of anaphylaxis is unpredictable, determining its incidence and characteristics is important for prevention and treatment [1, 2]. Although the incidence of anaphylaxis is different in each country, it is increasing worldwide. The frequency of anaphylaxis caused by food is increasing in adolescents and young adults [3–5].

In 2001–2010 analyzing the incidence of anaphylaxis in the United States increased annually from 36.8 to 46.6 per 100,000 population, increasing 4.3% per year and food-related by 9.8% per year [6].

In Korea, drugs were the most common cause of anaphylaxis in adults between 2007 and 2011, accounting for 46.5%, followed by food (24.2%) [1]. In the 2001 to 2007 survey, the incidence of anaphylaxis in children and adolescents in Korea was 0.7 to 1.0 per 100,000 people. Most of the causes were unknown (61.7%), and food (24.9%) was the second most common cause [7]. Subsequently, a multicenter analysis between 2009 and 2013 reported that food (74.7%) was the most common cause of anaphylaxis in children and adolescents [8].

Owing to the nature of the disease, most patients visit the emergency department (ED) first. More and more studies are publishing on patients with anaphylaxis to ED [9–12]. Children are a vulnerable group; hence, accurate diagnosis and analyses of the incidence and causes of anaphylaxis among children are of the utmost importance [12].

This study aimed to determine the incidence, causes, regional, and seasonal patterns of the disease by analyzing the characteristics of patients with anaphylaxis. Only anaphylaxis cases according to ICD-10 codes were investigated.

## Materials and methods

### Participants

Data on 147 EDs from the National Emergency Department Information System (NEDIS) from 2007 to 2013 were retrospectively analyzed. Among the patients who visited the ED, those with the primary diagnosis of anaphylaxis according to the *Tenth Revision of the International Classification of Diseases* (*ICD-10*) classification codes—T78.0 (anaphylactic shock due to adverse food reaction), T78.2 (anaphylactic shock, unspecified), T80.5 (anaphylactic shock due to serum), and T88.6 (anaphylactic shock due to the adverse effect of an appropriate drug or medication properly administered)—were included in the study [13].

To further understand the factors that trigger the occurrence of anaphylaxis, cases involving other external causes indicated by the lower classification codes T63 (toxic effect of contact with venomous animals) or X23 (contact with hornets, wasps, and bees) were classified as anaphylaxis cases. Initial clinical manifestations were analyzed by unified medical language system (UMLS) codes that correspond to signs or symptoms.

Incheon Medical Center Institutional Review Board waived the need for informed consent since the data was anonymized (115288-201610-HR-020-02).

### Statistical analysis

PASW Statistics version 18.0 (SPSS Inc., Chicago, IL, USA) was used for statistical analyses. Each variable was compared using the chi-square test.

Data from the Korean Statistical Information Service (KOSIS) were referenced for population statistics and population by age group [14]. Using this data as a denominator, the crude incidence rate and age-specific incidence rate were calculated. Poisson regression models were used to analyze the incidence trend of anaphylaxis and risk rate by gender, age-specific group, hospitalization, and region.

Statistical significance was defined as a *P* value of <0.05 using a two-tailed test.

## Results

### Study population

A total of 23,313 patients with anaphylaxis visited 147 EDs between 2007 and 2013, of whom 12,291 were men and 11,022 were women, with a male-to-female ratio of 1:0.90, with no significant difference (Table 1). Those aged 50–59 years had the highest rate of ED visits (22.8%), while those aged <1 year had the lowest rate of ED visits (0.7%; Table 1). Among the children and adolescents, those between ages 13 to 19 years visited the most (Table 1).

**Table 1:** Characteristics of ED visits with anaphylaxis, 2007–2013.

Characteristics	% (95% CI)
Total (n)	23,313 cases
Age, mean (SD) (years)	45.11 (19.18)
Gender	
Male	52.72 (52.08–53.36)
Female	47.28 (46.64–47.92)
Age group (years)	
< 12months	0.67 (0.57–0.79)
1~6	3.00 (2.79–3.23)
7~12	3.08 (2.86–3.31)
13~19	5.81 (5.52–6.12)
20~29	9.05 (8.69–9.43)
30~39	12.97 (12.54–13.40)
40~49	19.15 (18.65–19.66)
50~59	22.79 (22.25–23.33)
60~69	14.37 (13.92–14.82)
70~79	7.52 (7.19–7.87)
over 80 years	1.59 (1.43–1.76)
Outcomes	
ED discharge	77.27 (76.73–77.81)
Hospitalization	21.27 (20.74–21.80)
General ward	70.91 (69.63–72.18)
ICU	29.08 (27.82–30.37)
Deaths	0.15 (0.11–0.21)
Transfer	1.06 (0.93–1.19)
Others	0.09 (0.06–0.14)
Unknown	0.16 (0.11–0.22)

ED, emergency department; ICU, intensive care unit.

After treatment for anaphylaxis in the ED, 77.3% of patients were discharged (Table 1). Of the 4,958 inpatients, 70.9% and 29.1% were admitted in general wards and intensive care units (ICU), respectively (Table 1).

Of the total 23,313 cases, T78.2 was the most common at 80.8%, and T88.6 was 8.9% (Table 2).

**Table 2:** The number of cases of ICD-10 codes.

ICD-10 codes	Description	Cases (total 23,313) (%)
T78.2	Anaphylactic shock, unspecified	18,844 (80.8)
T88.6	Anaphylactic shock due to adverse effect of correct drug or medicament properly administered	2,070 (8.9)
T78.0	Anaphylactic shock due to adverse food reaction	960 (4.1)
X23	Contact with hornets, wasps and bees	757 (3.2)
T63.4	Venom of other arthropods	534 (2.3)
T80.5	Anaphylactic shock due to serum	115 (0.5)
T63.4, T88.6		13 (0.1)
T63.4, T80.5		7 (0.0)
T78.0, T88.6		6 (0.0)
T63.0	Snake venom	3 (0.0)
T63.4, T78.0		2 (0.0)
T63.9	Toxic effect of contact with unspecified venomous animal	1 (0.0)
T78.0, T80.5		1 (0.0)

Among the symptoms due to anaphylaxis at the time of ED visit, cutaneous and mucosal symptoms such as urticaria, facial swelling, and itching were the most common, accounting for 44.1% of all cases, followed by respiratory symptoms such as dyspnea and cough (19.2%). Cardiovascular symptoms such as hypotension, syncope, and chest pain occurred in 14.9% of patients; neurological symptoms such as dizziness, weakness, and headache occurred in 10.1%; and gastrointestinal symptoms such as diarrhea, nausea, and vomiting occurred in 6.3%. Of the total patients, 4.0% had other symptoms such as psychiatric symptoms, fever, and musculoskeletal pain.

### Annual trend of incidence, hospitalization, and fatalities in patients with anaphylaxis

**Fig 1:**
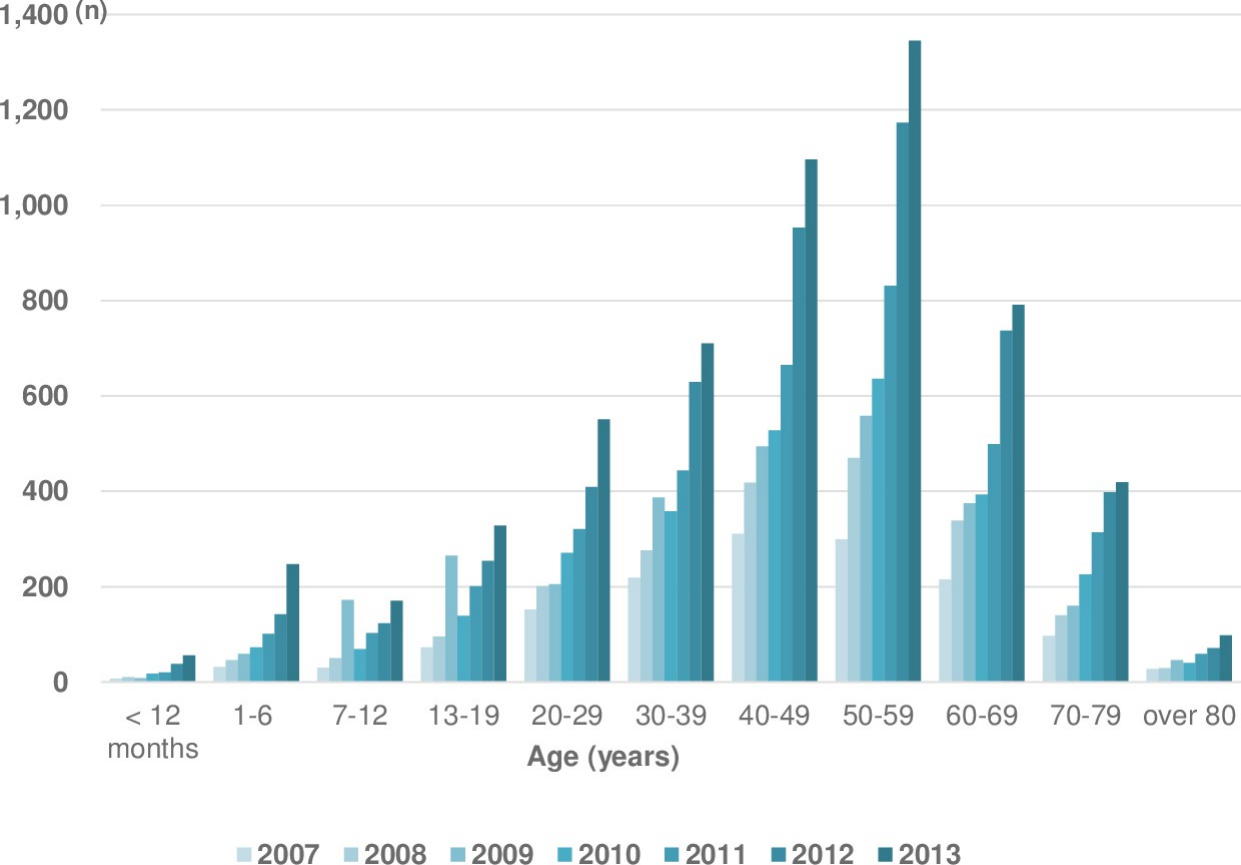
The emergency department visits with anaphylaxis by the age-specific group between 2007–2013 (*P* < 0.001).

The ED visits per 100,000 population increased from 3.0 in 2007 to 11.6 in 2013 (Table 3). Overall, the frequency of anaphylaxis emergency department visits increased by 1.237 times each year (95% CI 1.237–1.246, *P*<0.001) (Table 3 and Fig 1).

**Table 3:** Annual incidence rate with anaphylaxis by gender, age-specific group (years) and clinical outcomes.

	2007	2008	2009	2010	2011	2012	2013	Rate ratio (annual)*	95% CI	*P* value
Visits Per 100,000 population										
Total	3.0	4.2	5.5	5.6	7.1	9.9	11.6	1.237	1.237–1.246	<0.001
Male	3.3	4.6	5.8	5.7	7.3	10.5	12.2			
Female	2.8	3.8	5.3	5.5	7.0	9.2	10.9			
<12months	1.6	2.2	1.8	4.1	4.4	8.3	12.6	1.464	1.335–1.606	<0.001
1~6 years	1.1	1.6	2.5	2.6	3.7	5.1	8.8	1.402	1.344–1.462	<0.001
7~12	0.7	1.3	4.6	2.0	3.1	3.9	5.7	1.256	1.209–1.305	<0.001
13~19	1.6	2.0	5.5	2.8	4.1	5.3	7.0	1.216	1.183–1.251	<0.001
20~29	2.1	2.8	2.9	3.9	4.7	6.2	8.4	1.262	1.234–1.291	<0.001
30~39	2.5	3.2	4.6	4.3	5.4	7.7	8.9	1.226	1.203–1.249	<0.001
40~49	3.7	4.9	5.7	6.1	7.6	10.9	12.5	1.228	1.209–1.247	<0.001
50~59	5.2	7.8	8.8	9.5	11.6	15.6	17.3	1.202	1.185–1.219	<0.001
60~69	5.7	8.7	9.4	9.6	12.0	17.5	18.2	1.201	1.180–1.223	<0.001
70~79	4.5	6.2	6.7	8.9	11.8	14.0	14.0	1.208	1.178–1.239	<0.001
over 80 years	3.8	3.7	5.4	4.4	6.1	6.8	8.8	1.156	1.095–1.220	<0.001
Hospitalization	0.7	1.0	1.2	1.2	1.4	2.2	2.3	1.213	1.195–1.230	<0.001
Fatalities per 1,000,000 population										
	0.04	0.00	0.10	0.06	0.20	0.12	0.20	1.351	1.126–1.622	<0.001

* Rate ratio (annual) is the annual increase rate by Poisson regression analysis.

The frequency of ED visits increased significantly every year in all age-specific groups (Table 3).

The hospitalization cases after visiting the ED due to anaphylaxis per 100,000 population increased by 1.213 times a year (95% CI 1.195–1.230, *P*<0.001) (Table 3).

### The risk rate of ED visits and hospitalization due to anaphylaxis by age-specific group and region

The risk of visiting the ED due to anaphylaxis based on age-specific population was the highest in the 60–69 years old group compared to the under 1-year-old group (OR 2.302, 95% CI 1.962–2.702, *P*<0.001) (Table 4). The risk of hospitalization was 4.365 times higher in the 70–79 years old group (95% CI 1.938–9.830, *P*<0.001) (Table 4).

**Table 4:** The population-based annual risk rate of anaphylaxis visits and intensive care unit (ICU) hospitalization by gender and age-specific group.

Variables	OR	95% CI	*P* value
Incidence by gender			
Male	1		
Female	0.901	0.878–0.924	<0.001
Year			
2007	1		
2008	1.407	1.316–1.505	<0.001
2009	1.843	1.730–1.964	<0.001
2010	1.849	1.736–1.971	<0.001
2011	2.374	2.234–2.523	<0.001
2012	3.273	3.087–3.470	<0.001
2013	3.844	3.630–4.070	<0.001
Incidence by age group (years)			
<12 months	1		
1–6	0.721	0.607–0.857	<0.001
7–12	0.601	0.506–0.715	<0.001
13–19	0.804	0.682–0.948	<0.001
20–29	0.877	0.746–1.032	0.114
30–39	1.042	0.888–1.223	0.614
40–49	1.461	1.246–1.713	<0.001
50–59	2.149	1.834–2.519	<0.001
60–69	2.302	1.962–2.702	<0.001
70–79	1.874	1.591–2.206	<0.001
Over 80	1.096	0.910–1.321	0.335
Hospitalization by gender			
Male	1		
Female	0.914	0.865–0.966	0.001
Hospitalization by age group (years)			
<12 months	1		
1–6	0.227	0.081–0.637	0.005
7–12	0.258	0.095–0.697	0.008
13–19	0.352	0.147–0.846	0.020
20–29	0.574	0.249–1.322	0.192
30–39	0.871	0.384–1.976	0.742
40–49	1.535	0.683–3.452	0.300
50–59	2.991	1.335–6.701	0.008
60–69	4.256	1.897–9.546	<0.001
70–79	4.365	1.938–9.830	<0.001
Over 80	3.717	1.609–8.588	0.002
Incidence by region			
North Chungcheong	1		
Gyeonggi	1.725	1.563–1.904	<0.001
Seoul	2.016	1.827–2.225	<0.001
Busan	0.796	0.708–0.897	<0.001
South Gyeongsang	1.157	1.034–1.296	0.011
Incheon	1.097	.977–1.233	0.119
North Gyeongsang	1.641	1.469–1.832	<0.001
Daegu	1.368	1.220–1.534	<0.001
South Chungcheong	1.231	1.092–1.388	0.001
North Jeolla	2.313	2.070–2.585	<0.001
South Jeolla	1.812	1.614–2.034	<0.001
Daejeon	2.002	1.782–2.250	<0.001
Gangwon	3.335	2.992–3.718	<0.001
Gwangju	2.341	2.089–2.623	<0.001
Ulsan	1.222	1.063–1.403	0.005
Jeju	3.317	2.917–3.772	<0.001

**Fig 2:**
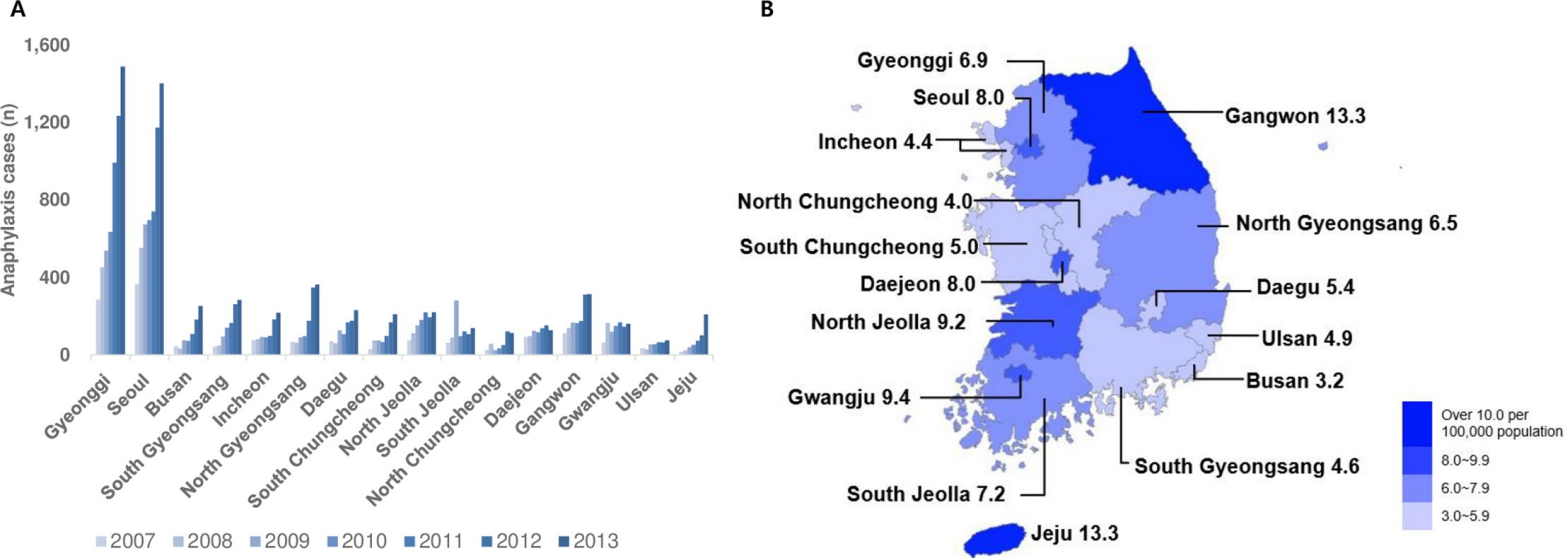
Regional distribution of anaphylaxis cases through 2007–2013. (A) Annual anaphylaxis cases by region. The names of the regions are in order of the highest mid-population. (B) The map of anaphylaxis cases per 100,000 populations. (Statistical Geographic Information Service (SGIS, http://sgis.kostat.go.kr)).

The regions with the highest number of patients with anaphylaxis between 2007 and 2013 were Gyeonggi (5,631 patients, 24.2%) and Seoul (5,612 patients, 24.1%), and these values were consistent with the order of the regions with the highest mid-populations. Although the patients who visited the ED due to anaphylaxis were high in the metropolitan area, Gyeonggi and Seoul, the risks of incidence per 100,000 population were high in Gangwon and Jeju (Table 4 and Fig 2).

### ICU hospitalization rate and inpatient mortality by age

**Fig 3:**
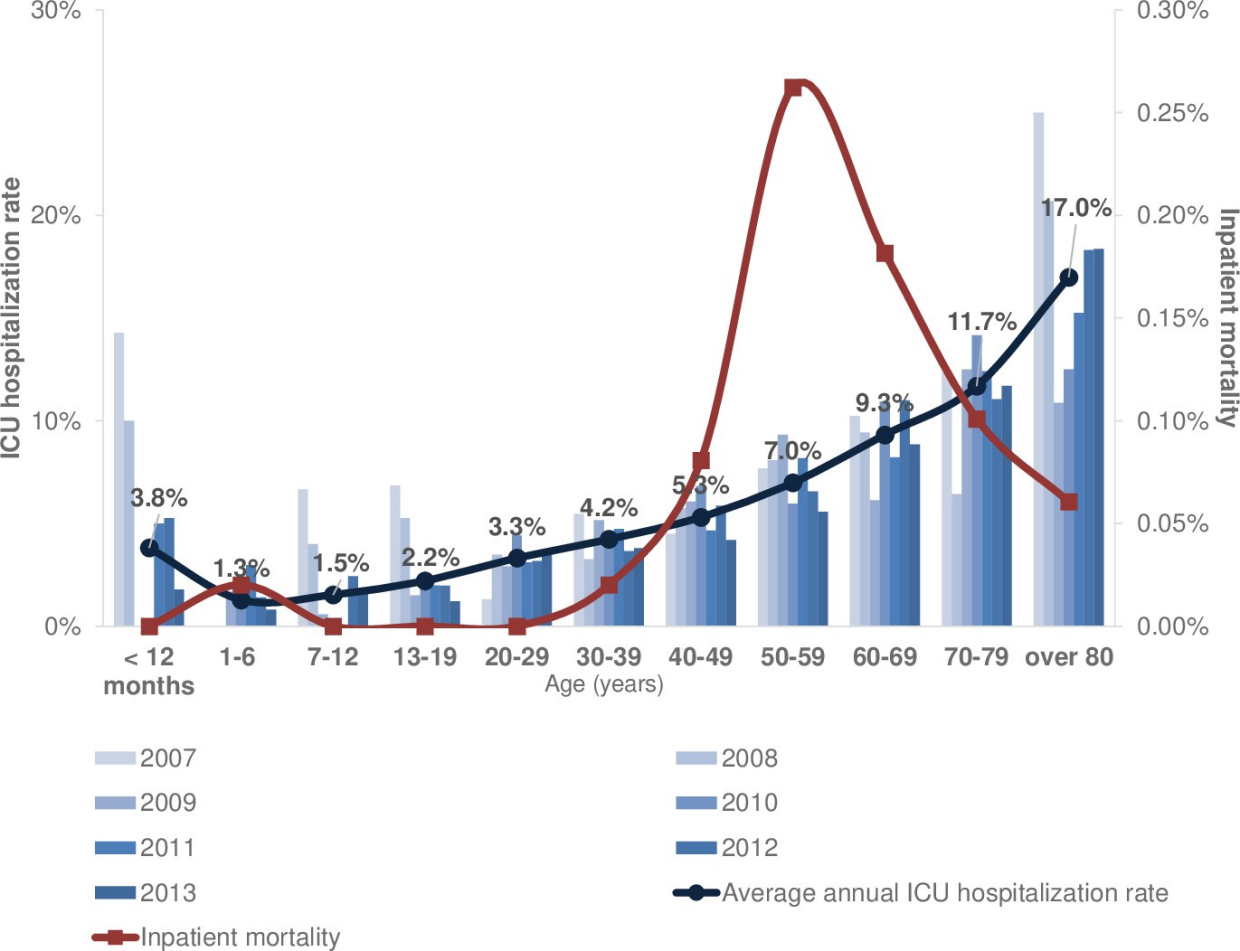
The intensive care unit (ICU) hospitalization rate and inpatient mortality by age group through 2007–2013.

Among children and adolescents who visited ED with anaphylaxis, those aged <1 year had the highest ICU hospitalization rate (14.3%) in 2007, which decreased annually up to 1.8% in 2013 (Fig 3). In patients aged <19 years, the ICU hospitalization rate also decreased each year. The ICU hospitalization rate was the highest in patients aged >80 years: 25.0% in 2007, 10.9% in 2009, and 18.4% in 2013 (Fig 3).

The mean ICU hospitalization rate during the 7-year investigation period was 3.8% in those aged <12 months and 1.5% in schoolchildren, which is less than half of that in infants (Fig 3). The mean ICU hospitalization rate during the investigation period was 17.0% in those aged >80 years (*P* < 0.001; Fig 3).

The inpatient mortality rate was highest in patients aged 50–59 years (0.26%) and 0.18% in those aged 60–69 years (Fig 3). Among the children, only one died.

### Distribution of monthly anaphylaxis incidence by year and age

**Fig 4:**
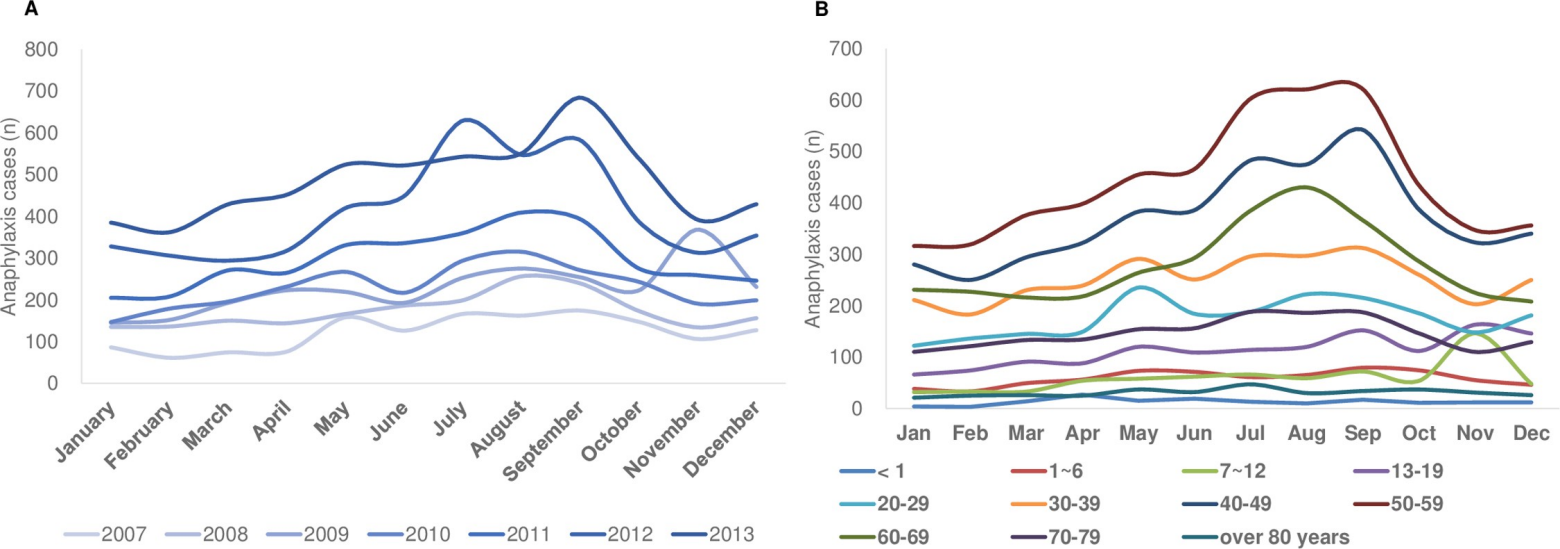
Seasonal emergency department visits with anaphylaxis, 2007–2013. (A) Annual anaphylaxis cases. (B) The monthly distribution by age group.

The number of patients with anaphylaxis with frequent visits to the ED was the highest in September (11.1%) and the lowest in February (6.0%; Fig 4A). The number of ED visits was the highest in July, August, and September (32.4%, *P* < 0.0001; Fig 4A). However, in 2009, the number of ED visits was the highest in November (Fig 4A). The number of ED visits due to anaphylaxis was the lowest in winter (January, February, and December).

By age, the 50–59-year age group had the most monthly ED visits, with the most visits in August and September (11.7%; Fig 4B). The 60–69-year age group had the most visits in August (12.84%), followed by July (11.50%; Fig 4B). The 40–49-year age group visited the most in September (12.1%; Fig 4B). In the 40–69-year age group, the most ED visits due to anaphylaxis occurred in the summer, between July and September.

In patients aged <6 years or >70 years, no significant difference was found in the monthly distribution of ED visits. In the 7–12-year and 13–19-year age groups, most visits occurred in November, at frequencies of 20.4% and 12.0%, respectively (Fig 4B). Most visits to the ED occurred in May for the 20–29-year age group (11.1%) and in September for the 30–49-year age group (10.3%; Fig 4B).

### The causes of anaphylaxis by *ICD-10* codes

**Fig 5:**
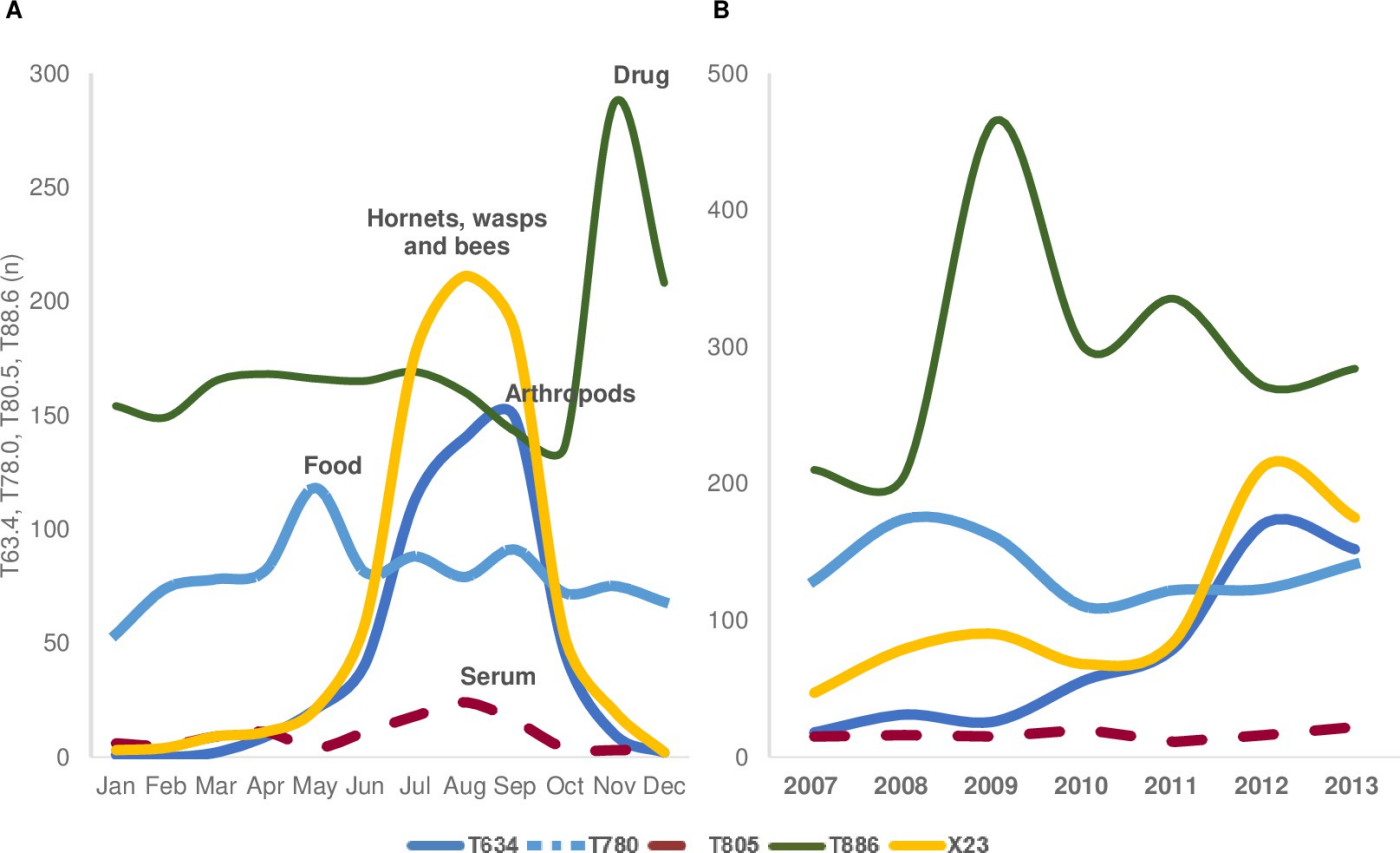
Monthly (A) and annual (B) distribution of the causes of anaphylaxis through 2007–2013. T63.4, venom of other arthropods; T78.0, anaphylactic shock due to adverse food reaction; T80.5, anaphylactic shock due to serum; T88.6, anaphylactic shock due to the adverse effect of an appropriate drug or medication properly administered; T78.2, anaphylactic shock, unspecified; X23, contact with hornets, wasps, and bees.

**Fig 6:**
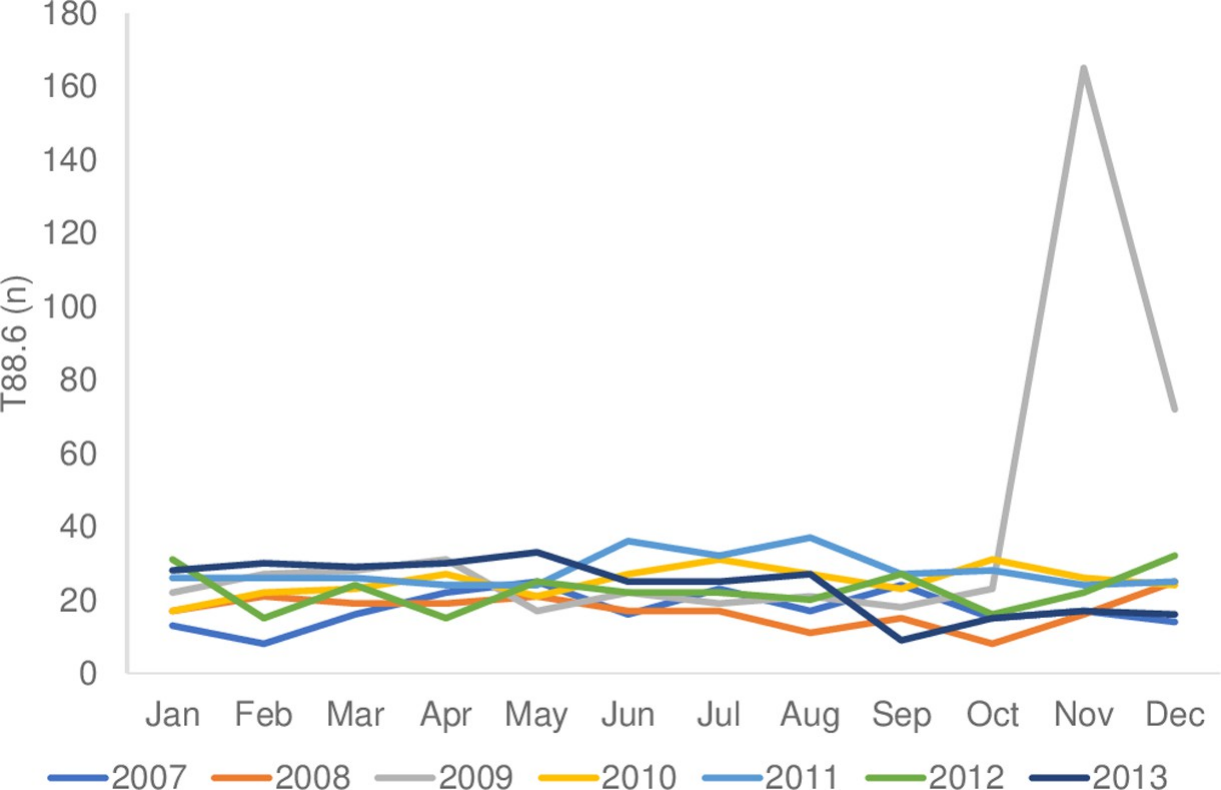
Monthly distribution of T88.6 through 2007–2013 (*P*<0.001). T88.6, anaphylactic shock due to the adverse effect of an appropriate drug or medication properly administered.

In the analysis of the monthly distribution of diagnostic codes, drug-related anaphylaxis (T88.6) occurred most frequently in November (Fig 5A). In 2009, 35.5% of cases occurred in November and 15.5% occurred in December (Fig 6). The most frequent causes of anaphylaxis were hornets, wasps, and bees, followed by arthropods, in summer. The number of cases involving arthropods as a cause started to increase in July and August, the summer months in Korea, and peaked in September (Fig 5A). Food-related anaphylaxis occurred most frequently in May (12.3%; Fig 5A).

Food-related anaphylaxis was the highest in 2008 (18.1%), and then showed a decreasing trend from 2010 (Fig 5B). The arthropods and bee stings were highest in 2012 (Fig 5B).

**Fig 7:**
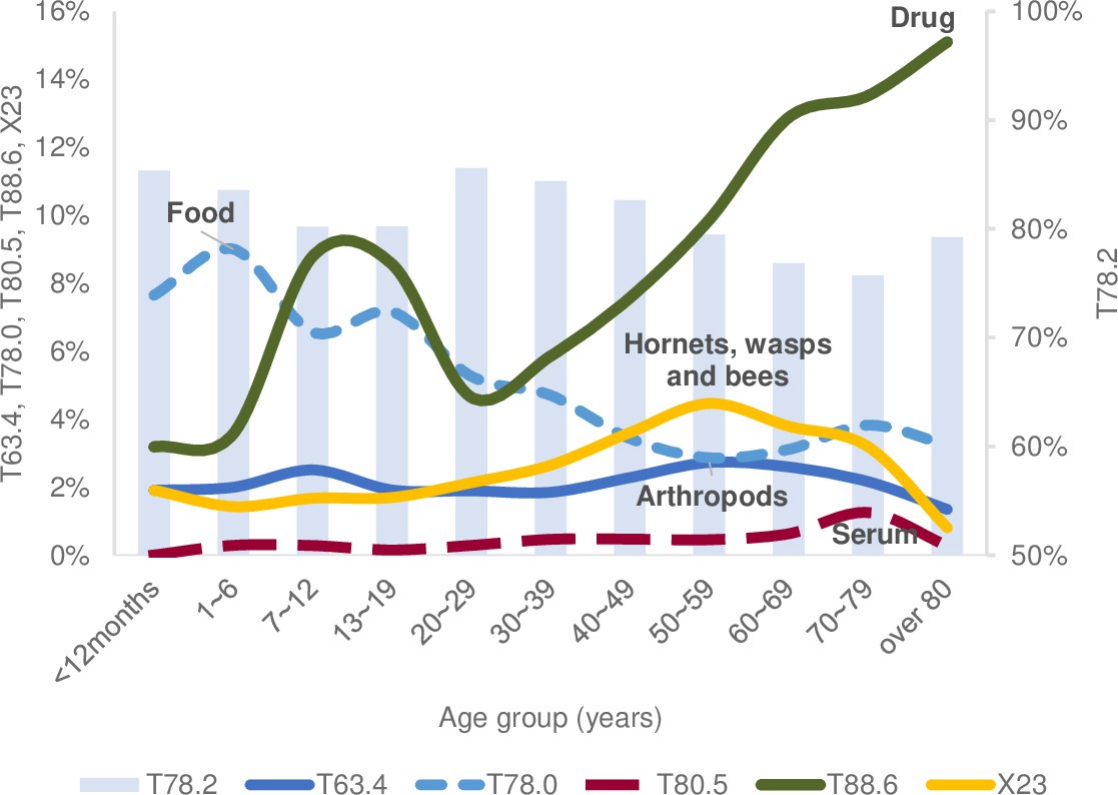
The causes of anaphylaxis within the age group through 2007–2013. T63.4, venom of other arthropods; T78.0, anaphylactic shock due to adverse food reaction; T80.5, anaphylactic shock due to serum; T88.6, anaphylactic shock due to the adverse effect of an appropriate drug or medication properly administered; T78.2, anaphylactic shock, unspecified; X23, contact with hornets, wasps, and bees.

In children aged >7 years and in adults, drugs were the most common cause of anaphylaxis (Fig 7). By the age of 19, food accounted for 30.4% of anaphylaxis, and after the age of 20, the frequency of food anaphylaxis gradually decreased.

Drug-related anaphylaxis was most common among those over the age of 80, accounting for 15.1%. The second most frequent cause was food (7.6% of patients aged <1 year and 9.0% of patients aged between 1 and 6 years).

The X23 (hornets, wasps, and bees) was the most common in the 40–69-year-old group, accounting for 69.4%. Among them, those in their 50s accounted for the most at 31.3% (Fig 7).

**Fig 8:**
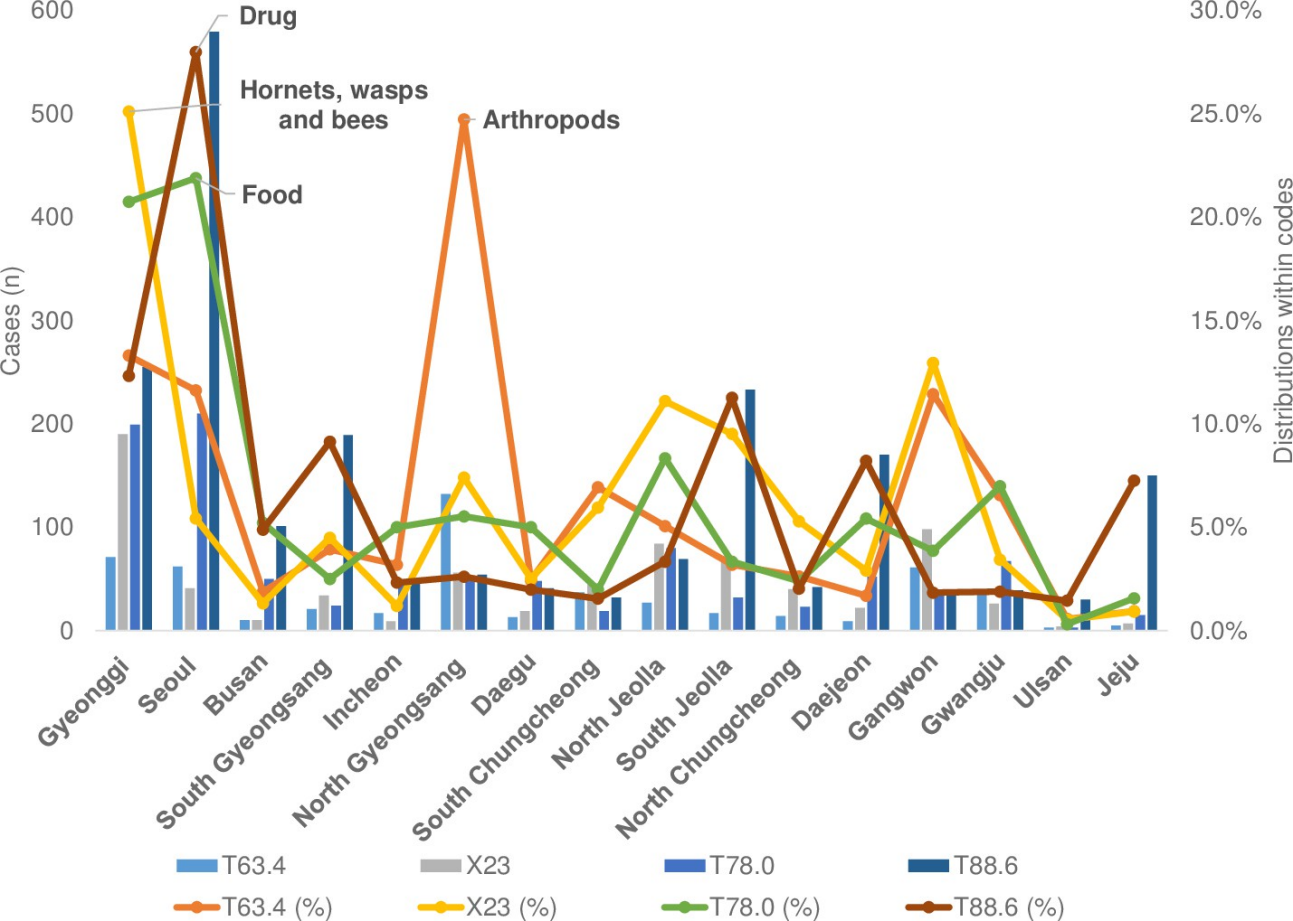
Regional distribution of anaphylaxis diagnostic codes through 2007–2013. The names of the regions are in order of the highest mid-population. T63.4 venom of other arthropods; T78.0, anaphylactic shock due to adverse food reaction; X23, contact with hornets, wasps, and bees; T88.6 (anaphylactic shock due to the adverse effect of an appropriate drug or medication properly administered).

Most cases of anaphylaxis caused by T88.6 (drug) occurred in 10 out of 16 administrative districts nationwide. However, X23 (hornets, wasps, and bees) was the most common in South Chungcheong, North Jeolla, and Gangwon. In North Gyeongsang, the most cases were due to T63.4 (arthropods), followed by X23 (Fig 8).

The regions with the most X23 (hornets, wasps, and bees) were Gyeonggi (25.1%) and Gangwon (12.9%), and the district with the most T63.4 (arthropods) was North Gyeongsang (24.7%) and Gangwon (11.4%) (Fig 8).

## Discussion

The incidence of anaphylaxis in Korea increased by 287% during the 7-year-investigation period, from 3.0 in 2007 to 11.6 per 100,000 people in 2013. This increase is considered significant compared with that reported in the United States between 2005 and 2014 [12].

We have suggested results for many variables related to the frequency of anaphylaxis in ED.

There are some differences in an epidemiological study due to physicians’ underdiagnosis of anaphylaxis, different guidelines between countries, and patient registration systems [15].

Physicians create missing cases by registering incorrect diagnostic codes or even classifying them as other disorders instead of anaphylaxis depending on the severity of the symptoms [16, 17].

In several studies on the increasing trend of anaphylaxis, it is critical to know that missing data can be a problem, potentially resulting in inaccurate statistical results.

The reason for the high increase of anaphylaxis in Korea, which is a part of Asia, seems to be due to the physicians’ awareness of the diagnosis or the rise in the number of actual patients with anaphylaxis owing to various allergens. The import of nuts, one of the causes of food anaphylaxis, increased significantly in Korea (226%) between 2003 and 2012 [18].

By age, the incidence of anaphylaxis in children aged <19 years was 12.6%, of which 8.9% were schoolchildren aged between 7 and 19, more than half of the incidence in children and adolescents (Table 1). In Korea, most of the causes of anaphylaxis in children aged <19 years were food. A survey by the Korean Health Insurance Review and Assessment Service, most anaphylaxis cases involved unknown (61.7%), followed by food (24.9%) [7, 8, 19, 20]. In 2008, food accounted for 65% of the causes in children aged <17 years, but between 2009 and 2013, this value increased to 74.7% in children aged <6 years [8, 19]. As a result of this study, 16.6% of children under the age of 6 had anaphylaxis caused by food, but it was not possible to identify causing specific foods. Drugs accounted for the largest number of cases (17.3%) of anaphylaxis in the 7–19-year age group, followed by food (13.7%).

In other Korean multicenter studies, the anaphylaxis in infants has increased, and 93.1% of infants aged <2 years experienced anaphylaxis due to food [8, 20]. In a report from the European Anaphylaxis Registry data between 2007 and 2015, anaphylaxis onset occurred at home in 46% of cases involving children and adolescents aged <18 years; food was the leading cause in 66% of cases, and the rate of emergency epinephrine treatment increased from 12% in 2011 to 25% in 2014 [21]. More active education and management are needed not only for students but also for pre-schoolers.

In 2009, the incidence of drug-related anaphylaxis was 35.5% in November and 15.5% in December, which was more pronounced than in other years (Fig 6). Although the frequency of vaccine-related anaphylaxis is extremely rare worldwide, the incidence of anaphylaxis related to the H1N1 pandemic influenza vaccine was high in 2009 [22–25]. Possible mediators are considered influenza mono-vaccine during the 2009 pandemic [22, 26]. Similarly, in Germany, the incidence of vaccine anaphylaxis increased after the 2009 pandemic [22]. When introducing a newly developed vaccine or drug, it is necessary to pay attention to the possible occurrence of anaphylaxis and should monitor the risk factors.

Oral allergy syndrome (OAS) can trigger anaphylaxis [27, 28]. In the UK, food-related anaphylaxis hospitalization was most common in June and reported as an effect related to some pollen season peaks [27]. Plant pollen is blown away by the wind and can travel hundreds to thousands of kilometers. Pollen level in Korea, especially birch, which is the cause of OAS, has high allergenicity and is mainly distributed in April-May [29]. Accordingly, we cautiously present the possibility of OAS as one of the reasons food-related anaphylaxis was the most prevalent in May.

Emergency visits of patients with anaphylaxis commonly occurred during summer, between July and September. Based on the results of the causal analysis, cases involving contact with hornets, wasps, and bees occurred most frequently in the summer, followed by those involving contact with arthropods. These results are like other studies in Korea, and it seems that there would have been many opportunities to expose poisonous animals and insects due to increased outdoor activities in summer [2, 25]. In Australia, the anaphylaxis caused by exposure to bees has not significantly increased since 1997 and has remained stable [30]. Also, insect-related anaphylaxis hospitalization was steady in the UK and USA [15]. However, most of the causes of death associated with anaphylaxis involve drugs (28%) and exposure to bees (13%), so it should be very vigilant [4, 30–32]. It is necessary to identify factors that cause insect-related anaphylaxis and preventive education. The increase in anaphylaxis by arthropods and bees can be inferred from the effects of changes in the ecological environment due to climate change or temperature rise [27, 33]. Repeated exposure to a trigger may be considered a real case, resulting in an increased frequency of anaphylaxis [9].

The regions with the most hornets, wasps, and bees were Gyeonggi (25.1%) and Gangwon (12.9%), and the areas with the most arthropods were North Gyeongsang (24.7%) and Gangwon (11.4%) (Fig 8). According to data from the Korea Forest Service in 2013, the regions with the most forest distribution in Korea are North Gyeongsang and Gangwon, consistent with the frequency distribution of arthropods [34]. When comparing the incidence by region with the forest map of Korea between 2011 and 2015, Gangwon had the largest increase in forest area, and Jeju and North Jeolla had the widest forest distribution [35]. These areas correspond to the regions with the highest number of patients with anaphylaxis per 100,000 people. In addition, drug-related anaphylaxis cases were the most common in most parts of the country, but in South Chungcheong, North Jeolla, and Gangwon, the diagnoses of anaphylaxis caused by a bee were the most common. Among these regions, North Jeolla and Gangwon are consistent with the areas in a study that reported that bee anaphylaxis was the most common [25]. Therefore, we can indirectly estimate that the regional differences in the incidence of anaphylaxis related to forest distribution and that arthropods, bees, and insect bites contributed to the causes of anaphylaxis. In the future, the frequency of anaphylaxis can be affected by changes in forest distributions or species due to climate change.

The prevalence of anaphylaxis was approximately 10 per 100,000 people before 2005, but subsequent studies have reported a prevalence of >50 per 100,000 people [32, 36]. In general, anaphylaxis caused by food is common in schoolchildren, and that caused by venom is common in adults aged 40–60 years [11, 37].

Anaphylaxis tended to increase every year and reached 32.2 episodes per 100,000 person-years in 2014 [1, 38]. In the past, anaphylaxis affected approximately 1%–2% of the population [39]. In the UK, the incidence of anaphylaxis increased 12-fold between 1991 and 2004. In a 1996–2005 cohort, the incidence of anaphylaxis per 100,000 people was reported to be 21.3 in those without asthma and 50.5 in those with asthma [40, 41]. In the US, the of anaphylaxis in EDs per 100,000 people increased from 14.2 in 2005 to 28.6 in 2014. The incidence per 100,000 people was 34 in France in 2015, 112.2 in Spain in 2012, and 17.6% in Canada between 2007 and 2012 [9, 11, 36, 42–44]. Japan in Asia had an incidence of 13.3 per 100,000 people in 2014, while China’s incidence of children under 18 years old increased from 2.46 in 2001 to 6.63 in 2015 [45, 46]. The frequency of anaphylaxis varies between countries, and it is more difficult to obtain accurate data [15]. The difference in the frequency of anaphylaxis is due to different anaphylaxis guidelines by countries, underdiagnosed coding problems, and difficulties in identifying cases occurring in the communities [15–17, 47]. Depending on the severity of symptoms, the diagnosis of anaphylaxis tends to be omitted or underdiagnosed, and many cases of pediatric anaphylaxis were registered incorrectly with another diagnosis, not anaphylaxis [16, 17].

Hospitalization rates also vary, particularly in the UK, where national guidelines recommend even hospitalization when a child is first diagnosed with anaphylaxis [15, 47, 48].

The hospitalization rate due to anaphylaxis tended to decrease from 24.0% to 20.2% from 2007 to 2013. But this value was still higher than that of France (14.2%), the US (15%), and Denmark (14.5%) in 2015 [6, 42, 49]. In children, the ICU hospitalization rate decreased every year and fluctuated in adults. This result suggests that the age-related differences might be due to differences in pathophysiology depending on the cause of anaphylaxis [37]. Besides, the ICU admission rate increased as the age increased; hence, age and comorbidity are considered risk factors that could affect the severity of the disease.

In children and adolescents, the ICU hospitalization rate was the highest in the <12-month age group (3.8%), and in schoolchildren was less than half of that in infants. It seems like a positive result of labelling food allergens began in 2003. It is necessary to provide food allergy education and management to infants under 12 months old.

In their 50s, anaphylaxis cases besides inpatient mortality were the most common. Moreover, the number of deaths among inpatients was the lowest in children and the highest in adults aged 50–59; prevention education on anaphylaxis according to age is necessary. Drugs accounted for 51.3% of all age groups in those 50 and older (Fig 7). Our study shows similar results that medicines account for the highest number of causes in adults as in the reports of anaphylaxis in other countries [15, 30, 31, 50]. However, another study in Korea, which had fewer participating hospitals than our result, reported more bee venom than drugs [25]. As described earlier, the causes of anaphylaxis may vary depending on the environment of each region. In the UK, comorbidities and concomitant medications were as risk factors for anaphylaxis [40]. Based on our results, physicians need to be aware of the drug-related anaphylaxis with increasing age, as the elderly population has variety of comorbid conditions.

This study has some limitations. Retrospective analysis of NEDIS data, we could not identify the various causes of anaphylaxis. It did not describe the medical history, laboratory test results, and adrenaline usage.

In England, the National Health Service (NHS) hospital data of early 1990s analysis reported that the rate of anaphylaxis hospitalization was higher in the rural area and affluent one [51]. We could not analyze the difference between rural and urban because the raw materials did not provide records on administrative sub-districts.

In analyzing data, results may differ depending on the definition of anaphylaxis and severity assessments used [9, 31, 52]. The severity could not be consistently classified in this study, as the guidelines or severity classification used by the physicians to diagnose anaphylaxis in the ED were unknown.

Nevertheless, the data used in this study have relatively little miscoding, no distortion for reimbursement of insurance costs, and high reliability.

This study presents the data of patients with anaphylaxis who visited 147 EDs; hence, the number of cases might be higher than this study. We found that the patterns of ED visits of patients with anaphylaxis differed by season, region, and age between 2007 and 2013 and that the annual number of ED visits of patients with anaphylaxis significantly increased.

Anaphylaxis is unpredictable as various risk factors may be involved. Initial use of adrenaline and avoidance of risk factors is crucial to lowering mortality [31, 48, 52, 53]. Medical practitioners need to be aware of the seriousness of anaphylaxis and need to provide an accurate diagnosis. More active treatment can prevent anaphylaxis.

In summary, patients with anaphylaxis who visited EDs in Korea between 2007 and 2013 have increased. Most ED visits due to anaphylaxis occurred between July and September, and the most frequent cause in most parts of the nation, except in some areas, was drugs. Between July and September, hornets, wasps, and bees were the most frequent cause, followed by arthropods. Food was the most common cause under the age of 6, and drugs in those >7 years.

## Conclusions

In Korea, based on ICD-10 codes, the number of ED visits due to anaphylaxis is increasing, and the incidence of anaphylaxis varies by region, season, and age. Consequently, the results can contribute to the prevention of anaphylaxis, improvement in disease awareness, and suggestion of management policies. Besides, pattern analysis of incidence will help establish various hypotheses for anaphylaxis research as well as these might be representative data in Korea.

These can serve as a basis for building an alarm system that predicts regional and seasonal anaphylaxis frequency and will help ensure prompt and appropriate treatment.
